# Exploring the implementation of an SMS-based digital health tool on maternal and infant health in informal settlements

**DOI:** 10.1186/s12884-024-06373-7

**Published:** 2024-03-27

**Authors:** Sharon Ochieng’, Nisha Hariharan, Timothy Abuya, Chantalle Okondo, Charity Ndwiga, Charlotte E. Warren, Anneka Wickramanayake, Sathyanath Rajasekharan

**Affiliations:** 1 Jacaranda Health, Jabavu Gate 788, Jabavu Road, Nairobi, 52595-00100 Kenya; 2 Population Council, Nairobi Kenya. Avenue 5, 3 Floor, Rose Avenue, Nairobi, 17643-00500 Kenya; 3 Population Council, Washington, DC. Suite 280, 4301 Connecticut Ave NW, Washington, DC, 20008 US

**Keywords:** Digital health, Maternal health, Kenya, Informal settlements, Antenatal care, Postnatal care

## Abstract

**Background:**

The rapid urbanization of Kenya has led to an increase in the growth of informal settlements. There are challenges with access to maternal, newborn, and child health (MNCH) services and higher maternal mortality rates in settlements. The Kuboresha Afya Mitaani (KAM) study aimed to improve access to MNCH services. We evaluate one component of the KAM study, PROMPTS (Promoting Mothers through Pregnancy and Postpartum), an innovative digital health intervention aimed at improving MNCH outcomes. PROMPTS is a two-way AI-enabled SMS-based platform that sends messages to pregnant and postnatal mothers based on pregnancy stage, and connects mothers with a clinical help desk to respond and refer urgent cases in minutes.

**Methods:**

PROMPTS was rolled out in informal settlements in Mathare and Kawangware in Nairobi County. The study adopted a pre-post intervention design, comparing baseline and endline population outcomes (1,416 participants, Baseline = 678, Endline = 738). To further explore PROMPTS's effect, outcomes were compared between endline participants enrolled and not enrolled in PROMPTS (738 participants). Outcomes related to antenatal (ANC) and postnatal (PNC) service uptake and knowledge were assessed using univariate and multivariate linear and logistic regression.

**Results:**

Between baseline and enldine, mothers were 1.85 times more likely to report their babies and 1.88 times more likely to report themselves being checked by a provider post-delivery. There were improvements in moms and babies receiving care on time. 45% of the 738 endline participants were enrolled in the PROMPTS program, with 87% of these participants sending at least one message to the system. Enrolled mothers were 2.28 times more likely to report completing four or more ANC visits relative to unenrolled mothers. Similarly, enrolled mothers were 4.20 times more likely to report their babies and 1.52 times more likely to report themselves being checked by a provider post-delivery compared to unenrolled mothers.

**Conclusions:**

This research demonstrates that a digital health tool can be used to improve care-seeking and knowledge levels among pregnant and postnatal women in informal settlements. Additional research is needed to refine and target solutions amongst those that were less likely to enroll in PROMPTS and to further drive improved MNCH outcomes amongst this population.

**Supplementary Information:**

The online version contains supplementary material available at 10.1186/s12884-024-06373-7.

## Introduction

Kenya is home to a rapidly growing population. According to the World Bank, annual urban population growth in Kenya was 3.7% in 2021 [1]. This growth has led to the proliferation of informal settlements (or slums), especially in Nairobi. Nairobi has the highest rate of urbanization in the country with 60 percent of the population residing in informal settlements [2]. The growth of urban informal settlements is characterized by overcrowding, social and economic marginalization, poor environmental conditions, and insecurity. Access to maternal, newborn, and child health (MNCH) services is more challenging for poor populations living in urban settings because of poverty, low levels of education, unemployment, younger maternal age, low social integration and support, socio-cultural taboos, and having displaced, refugee, or migrant backgrounds [3]. Given the contextual drivers of poor health in informal settlements, there is a need for innovative and targeted solutions that reach marginalized sub-populations and improve access to information and high-quality health care [4, 5]. This study attempted to test the implementation of one intervention in two diverse informal settlements—Kawangware and Mathare—in Nairobi County, Kenya, where almost 60,000 vulnerable women and children live and the maternal mortality rate is almost twice the national average (362/100,000 live births) [6].

**Fig. 1:**
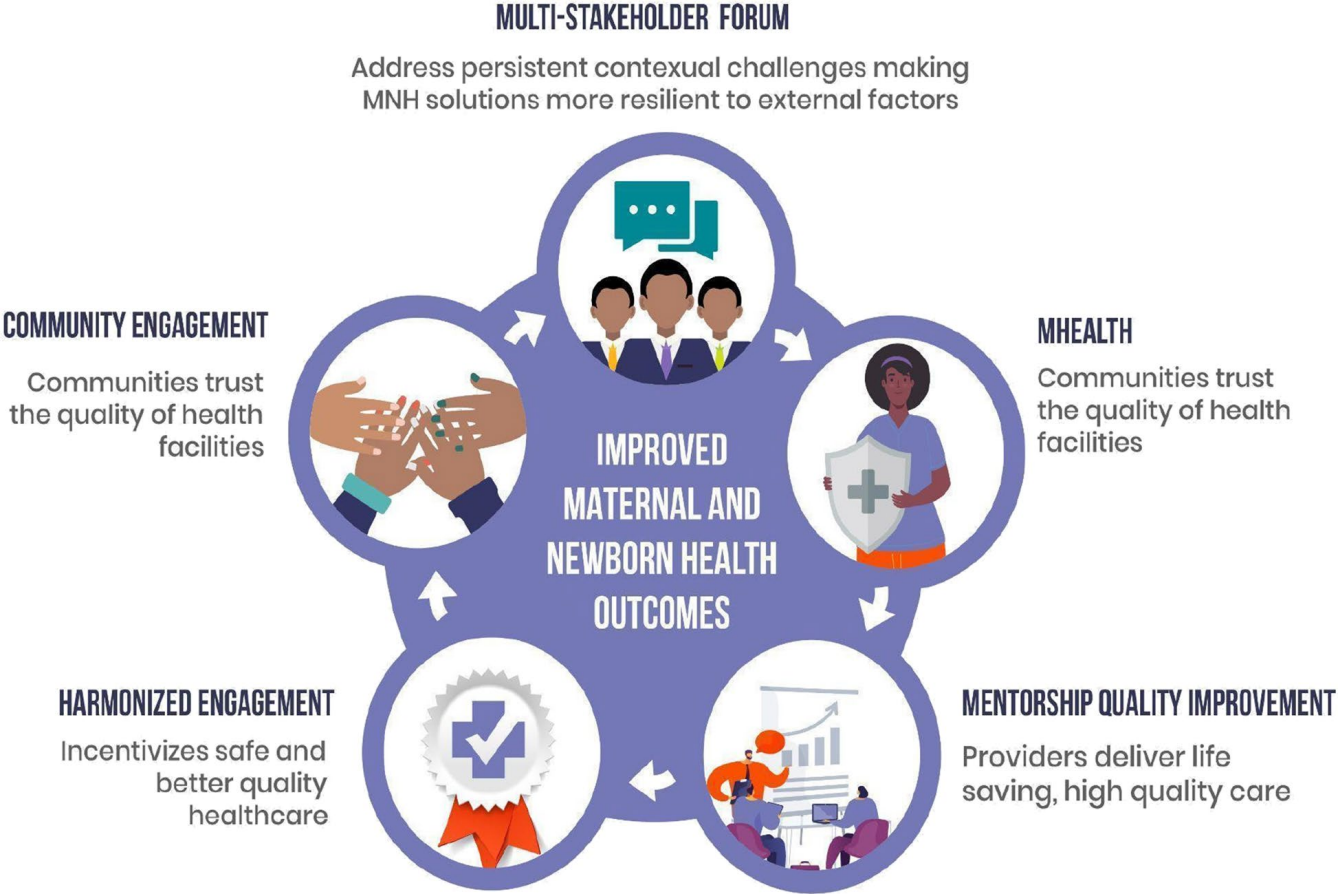
Quality Ecosystem

The Kuboresha Afya Mitaani (KAM) project aimed to improve MNCH outcomes for women in informal settlements of Nairobi. The project’s goal was to demonstrate how innovative approaches to tackling complex health challenges in urban settings can be integrated and replicated in other urban health contexts across Sub-Saharan Africa. Recognizing the importance of participatory approaches to strengthening often-fragmented informal health systems, the study was built around a ‘Quality Ecosystem’ (Fig. 1), integrating typically siloed actors in the quality of care space around solutions to MNCH challenges that mutually reinforce one another. The study was implemented by Jacaranda Health, Population Council, Nairobi City County government (then Nairobi Metropolitan Services—NMS), Sanergy, and the Berkeley Air Monitoring Group.

The high prevalence of mobile phones [7] in Low Middle Income Countries (LMICs) presents an opportunity to determine how to use this communication channel to advance MNCH. Many initiatives have attempted to leverage mobile phone reach for health, however, studies have shown that successful implementation of mHealth interventions in LMICs is often hampered by a number of issues: a lack of government buy-in and ownership, inadequate infrastructure and equipment, a lack of human resources and skills, inadequate legislation, an unstable or insufficient power supply, a lack of funding for sustainability, and weak evaluation mechanisms [8, 9].

Studies previously undertaken in Kenya primarily centered around the mHealth strategic domain, with a focus on primary care. However, only a few initiatives were executed in marginalized regions, despite exhibiting heightened healthcare requirements [10]. Despite the limited empirical evidence [8], it is noteworthy that SMS messaging has demonstrated an impact on the uptake of maternal and neonatal health interventions [8]. These projects strategically leverage the widespread penetration of mobile phones in low- and middle-income countries (LMIC) [11–13].

This study evaluated one component of the KAM project: PROMPTS (Promoting Mothers through Pregnancy and Postpartum). This paper presents the results of implementation research to evaluate the effectiveness and utilization of the PROMPTS program in the informal settlements of Mathare and Kawangware. PROMPTS is a two-way telephone messaging mHealth platform that empowers individuals to seek maternal, newborn and infant health care at the right time from pregnancy to the postnatal period. Research shows that mobile health (mHealth) interventions are increasingly recognized as tools for the delivery of health education and eliciting changes in care-seeking behaviors and MNCH outcomes [14]. In Kenya, nearly 89% of the population and 63% of the population in the lowest wealth quintile have access to a mobile phone [15], suggesting that mHealth interventions may be able to reach a large portion of the population. Similarly, PROMPTS’ goal is to reduce maternal and newborn deaths caused by delays in seeking health care.

A pre-post study design was used to understand the characteristics of PROMPTS participants in informal settlements and the effect of the PROMPTS program on (1) maternal and newborn care seeking, (2) maternal knowledge of complications and newborn caretaking practices, and (3) childhood immunization uptake. This research builds upon previous PROMPTS programming to evaluate if it can be adapted to improve maternal, newborn, and infant care seeking and knowledge in informal urban settings [16].

## Methods

### Study design

This implementation research study used a quasi-experimental design with a pre-post analysis to measure the implementation of PROMPTS within the larger KAM implementation research project. Comparisons were made between baseline and endline populations to understand the broader KAM influence on care-seeking and knowledge outcomes. To further isolate the effect of PROMPTS in informal settlements, outcomes were subsequently compared amongst endline participants who were enrolled in PROMPTS and those who were not enrolled in PROMPTS. Baseline (pre-intervention) data were collected from the two informal settlements in Mathare and Kawangware from October to November 2020. The PROMPTS intervention was subsequently launched in November 2020. Endline (post) data were collected in August 2022 for a month. Data were collected through community-level household surveys.

### Intervention overview

Jacaranda Health developed its Artificial Intelligence (AI)-enabled mHealth platform, PROMPTS, in 2017, with the aim of empowering users with information to seek care at the right time and place from pregnancy to the postnatal period. Mothers who enroll in PROMPTS receive a sequence of rigorously tested informative messages linked to gestational age, including (1) appointment reminders for antenatal care (ANC), postnatal care (PNC), and infant immunization; (2) follow-up after each facility check-up; and (3) information about danger signs, early childhood development, breastfeeding, complementary feeding, and infant care. In addition, users can send in questions free of charge which are responded to by an AI-enabled helpdesk. PROMPTS is already active in 20 counties across multiple geographic areas in Kenya.

Users were enrolled in PROMPTs during their first pregnancy-related or postpartum visit upon consenting to this at one of Jacaranda Health’s networks of hospitals or clinics or through self-enrollment via referrals from their peers. Information captured at enrollment included the user’s phone number, enrollment facility (if applicable), expected delivery date, and language preference (Swahili or English). The user received messages throughout the rest of the pregnancy and postpartum period from the user’s county government.

PROMPTS users were polled on a monthly basis to rate their facilities’ quality of care and provide feedback on experience (disrespect and abuse, consent) and technical quality (i.e. blood pressure taken, iron/folate tablets given). This feedback was aggregated to create a simple ‘experience scorecard.’ Information was discussed with facilities on a monthly basis and fed into a facility-client experience dashboard.

PROMPTS was adapted for the KAM study population by reviewing standard PROMPTS messages through focus groups discussions and reviews by Nairobi County and sub-county representatives. The PROMPTS platform was deployed in study facilities from November 2020 and targeted facilities that were likely points of care seeking for mothers living in Mathare and Kawangware. Facilities were selected based on volume of clients (ANC, maternity, PNC), government registration status, and geographic location/proximity to mothers in the target population.

### Study population

The intervention focused on women aged 15–49 years who were currently pregnant or had given birth within the last year (subcategorized as caregivers of children 0–6 months and 7–11 months). Households were included only if the woman intended to stay in the locality for the next six months.

### Sample size and sampling methodology

A modified Lot Quality Assurance Sampling (LQAS) approach was used to sample participants at baseline and endline. LQAS has been adapted to evaluate public health interventions and services, especially in lower- and middle-income countries [17]. Evidence shows that LQAS is a robust sampling design to identify general program coverage or communities having inadequate service coverage. A sampling frame was generated using the World Health Organization (WHO)’s LQAS guide [17].

The sampling frame was generated via a household listing. This activity was done using community health volunteers (CHVs) in collaboration with the Nairobi County Department of Community Health, from September 2020 at baseline and July 2022 at endline. The goal was to help identify households with pregnant women and caregivers of young infants who met the study population criteria. CHVs were used because they were permitted to engage with communities during the COVID-19 pandemic and due to their knowledge and connection with the community.

The study team first trained Community Health Extension Workers (CHEWs) virtually on the household listing process. The CHEWs, who supervise CHVs, trained them to complete the listing survey during routine household visits for those who provided consent.

Administratively, both informal settlements are organized into villages with Mathare having 13 villages while Kawangware has 9 villages. The listing form provided information on the number of pregnant women and caregivers aged 15–49 years who have infants 0–11 months in each sample village. The information captured included: location, CHV details, and household details (e.g., name of household head, contact information, and category of eligible participant). If the CHV identified two eligible participants in one household, they were listed together but would be interviewed separately. CHVs captured household data on paper or electronically via Open Data Kit (ODK) version 1.2.2.

A total of 6,429 households at baseline, and 8,088 at endline were listed. Households were from four villages in Kawangware (Kawangware, Kabiro, Gatina, Ngando) and six villages in Mathare (Mlango Kubwa, Hospital, Kiamaiko, Mabatini, Ngei and Huruma). The selection of villages was based on budget and advice from the Nairobi Department of Community Health. To implement the LQAS sampling procedure, Kawangware was subdivided into four villages and Mathare into six villages called Supervision Areas (SAs) or villages with at least a dedicated study health facility.

For each village (supervision area) three lots of samples were randomly selected: pregnant women, caregivers of young infants 0–6 months and 7–11 months. Of the listed households, 2,006 housed pregnant women; 2,406 housed caregivers of young infants 0–6 months; and 2,060 housed caregivers of infants 7–11 months at baseline and endline. A sample of 19 respondents from each of the lots provided acceptable levels of statistical error (90–95% confidence level $\alpha \le 5\%, \beta \le 20\%)$ similar to other LQAS studies [18, 19]. Each lot was oversampled by 20–30% to give the study team a margin of error for picking additional cases in the same lot in the event that the respondents could not be reached by phone, leading to 25 households being sampled per village and per lot. Approximately, 798 households were sampled at baseline and 938 households were sampled at endline, meeting the targeted sample size requirements.

### Data collection

Due to the COVID-19 pandemic, restrictions on movement in Nairobi and concerns related to the spread of the virus, in-person surveys were not possible at baseline. For this reason, the study was designed as a phone-based survey. To integrate with the household survey data, responses were recorded using ODK. The data collection entailed submission of the same data in real-time back to the central data collection point. A pilot was conducted to give the survey team a chance to amend the mobile system where necessary. The survey covered seven modules: sociodemographic information, sources of health information, ANC, labor and delivery, PNC, access to WASH services, and household assets and amenities. The endline survey included additional questions on exposure and experiences using the interventions.

Research assistants (RAs) were instructed to briefly introduce the study to the head of household and, if they agreed to participate, the eligible woman (pregnant or caregiver of a young infant) was asked to give consent and interviewed. RAs completed between one to six interviews per day each averaging one hour and made multiple attempts to call phone numbers and establish if women were still eligible to participate. Data quality measures were implemented: two RAs would conduct daily back-checks through call-backs for 10–20% of the completed interviews. The back checks were used to confirm that the study participant in the target group had spoken to an RA, and that the responses captured were accurate.

### Data analysis

Data were analyzed in STATA 15 (2009 StataCorp, College Station, TX). Descriptive data were produced by running Pearson’s chi2 tests for categorical variables and student t-tests for continuous variables. PROMPTS system data was used to link phone numbers provided during the household survey to enrollment records and user activity. Outcomes related to ANC and PNC services uptake, general knowledge of ANC and PNC care (e.g., danger signs during pregnancy and postpartum, newborn care practices) were compared between 1) participants surveyed at baseline and endline (pre-post), and 2) participants who were enrolled in the PROMPTS program versus those who were not enrolled in the PROMPTS program. Binary outcomes were evaluated using univariate and multivariate logistic regression models controlling for potential confounders. Continuous outcomes were evaluated using univariate and multivariate linear regression models controlling for potential confounders. Potential confounders included were age, wealth, marital status, sub-county, participant type, education, and employment status. The adjusted odds ratio (aOR) is presented in this paper which controls for potential confounders. Logistic regression was also used to understand characteristics of participants who were more likely to be enrolled in PROMPTS and engage with the PROMPTS platform. An α of 0·05 was assumed for all statistical tests of significance.

Asset-based measures (often called a wealth or asset index) were used to generate wealth quintiles. Information on ownership of a range of durable assets (e.g., car, refrigerator, and television), housing characteristics (e.g., material of dwelling floor and roof and main cooking fuel) and access to basic services (e.g., electricity supply, water supply and sanitation facilities) was collected. This data was used to construct an asset index. A decision was made to assign each indicator equal weights. A sum of indicators each household possesses was analyzed using Principal Components Analysis (PCA), a data reduction technique that seeks to establish the correlations between the indicators to generate a set of uncorrelated principal components, implicitly gives equal value in terms of socio-economic status (SES).

## Results

### Pre-Post Comparison Results

#### Participant characteristics

**Fig. 2:**
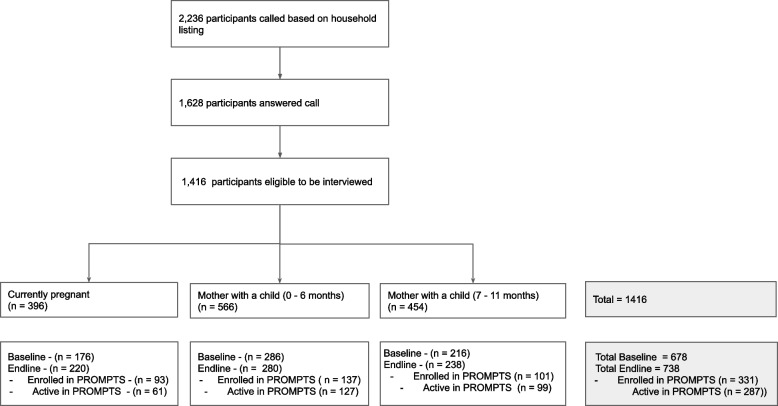
.

The pre-post analysis included 1,416 participants who were pregnant or had a newborn (0 – 11 months old) from Mathare and Kawangware in Nairobi County. There were 678 and 738 participants surveyed at baseline and endline, respectively (Table 1). The average age of participants was 27 years. The majority of participants had received secondary education or higher (61% (416) and 66% (490) for baseline and endline, respectively), and were unemployed (64% (434) and 68% (503) for baseline and endline, respectively). There was a statistically significant difference between the baseline and endline participants in terms of socioeconomic status. The endline population relative to baseline population had a higher proportion of wealthier participants, 68% (499) vs. 52% (351) of participants. Figure 2 provides an overview of the participants surveyed at baseline and endline, and enrollment and activity into the PROMPTS program.

**Table 1:** Baseline and endline demographic characteristics of study participants

Factor	Level	Baseline	Endline	*p*-value
		*n* (%)	*n *(%)	
N		678	738	
Participant type	Currently Pregnant	176 (26.0%)	220 (29.8%)	0.18
	Have a child 0-6 months	286 (42.2%)	280 (37.9%)	
	Have a child 7-11 months	216 (31.9%)	238 (32.2%)	
Age		27.4 (6.0)	27.8 (6.0)	0.21
Age distribution	15-19	42 (6.2%)	38 (5.1%)	0.069
	20-24	203 (29.9%)	216 (29.3%)	
	25-29	190 (28.0%)	237 (32.1%)	
	30-34	155 (22.9%)	132 (17.9%)	
	above 35	88 (13.0%)	115 (15.6%)	
Marital status	Married	453 (66.8%)	506 (68.6%)	0.48
	Single	225 (33.2%)	232 (31.4%)	
Education	Primary (no certificate/incomplete)	262 (38.6%)	248 (33.6%)	0.054
	Secondary ‘O’/ ‘A’ level (no certificate/incomplete)	336 (49.6%)	377 (51.1%)	
	College/Tertiary (certificate /complete)	80 (11.8%)	113 (15.3%)	
Wealth	Less Wealth	327 (48.2%)	239 (32.4%)	<0.001
	Higher Wealth	351 (51.8%)	499 (67.6%)	
Employment Status	Employed	244 (36.0%)	235 (31.8%)	0.1
	Unemployed	434 (64.0%)	503 (68.2%)	
Ward		7.7 (8.2)	7.1 (7.2)	0.21
	Dagoretti	270 (39.8%)	312 (42.3%)	0.35
	Mathare	408 (60.2%)	426 (57.7%)

### Antenatal knowledge and care-seeking

Outcomes related to ANC care seeking and maternal knowledge related to pregnancy and delivery complications were assessed amongst participants who were pregnant or had an infant between 0 to 6 months (Tables 2 and 3). ANC care-seeking was extremely high at baseline, with 93% of participants reporting attending ANC care. There were no statistically significant differences found between baseline and endline participants. There were also no significant differences between baseline and endline participants with respect to the proportion of participants with at least four ANC visits and reported knowledge levels (Tables 2 and 3). Although there was no change in the proportion of participants who reported knowing any signs of complication during pregnancy (78% at baseline and endline, Tables 4 and 5), there was a slight decrease in the mean number of pregnancy complications that were reported (2.5 versus 2.4, *p*-value = 0.01, Tables 6 and 7).

**Table 2:** Part 1: ANC care seeking amongst baseline and endline participants

	Baseline		Endline					
	*N*	*n* (%)	*N *	*n *(%)	Unadjusted OR	*p*-value	Adjusted OR^1^	*p*-value
Proportion attending ANC care	461	428 ( 93% )	498	459 ( 92% )	0.91	0.69	0.93	0.77
Proportion with at least 4 ANC visits, amongst those who attended ANC (Limited to mothers with a child aged 0-6 months)	277	198 ( 71% )	276	201 ( 73% )	1.07	0.72	0.96	0.84

**Table 3:** Part 2: ANC care seeking amongst those enrolled in PROMPTS and not-enrolled in PROMPTS at endline

	Endline - Not Enrolled		Endline - Enrolled					
	*N *	*n* (%)	*N*	*n* (%)	Unadjusted OR	*p*-value	Adjusted OR^1^	*p*-value
Proportion attending ANC care	268	239 ( 89% )	230	220 ( 96% )	2.67**	0.01	2.28*	0.05
Proportion with at least 4 ANC visits (Limited to mothers with a child aged 0-6 months)	140	97 ( 69% )	136	104 ( 76% )	1.44	0.18	1.38	0.25

1. Controlled for age, wealth, marital status, sub-county, participant type, education, and employment status

* *p*<0.05, ** *p*<0.01, *** *p*<0.001

**Table 4:** Part 1:  Maternal knowledge of danger signs during pregnancy and post delivery amongst baseline and endline participants (Logistic regression results)

	Baseline		Endline					
	*N *	*n* (%)	*N *	*n *(%)	Un-adjusted OR	*p*-value	Adjusted OR^1^	*p*-value
Proportion of mothers who reported knowing any signs of complications during pregnancy	461	361 ( 78% )	500	390 ( 78% )	0.98	0.91	0.92	0.6
Proportion of mothers who reported knowing any danger signs during delivery	461	215 ( 47% )	500	253 ( 51% )	1.17	0.22	1.17	0.24

**Table 5:** Part 2: Maternal knowledge of danger signs during pregnancy and post delivery amongst participants enrolled in PROMPTS and not-enrolled in PROMPTS at endline

	Endline - Not Enrolled		Endline - Enrolled		Un-adjusted OR	*p*-value	Adjusted OR^1^	*p*-value
	*N *	*n* (%)	*N *	*n* (%)				
Proportion of mothers who reported knowing any signs of complications during pregnancy	270	193 ( 71% )	230	197 ( 86% )	2.38***	0	2.20**	0
Proportion of mothers who reported knowing any danger signs during delivery	270	127 ( 47% )	230	126 ( 55% )	1.36	0.08	1.28	0.2

1. Controlled for age, wealth, marital status, sub-county, participant type, education, and employment status

* *p*<0.05, ** *p*<0.01, *** *p*<0.001

**Table 6:** Part 1:  Maternal knowledge of danger signs during pregnancy and post delivery amongst baseline and endline participants (amongst those who reported 'yes' for each respective category,  linear regression results)

	Baseline		Endline					
	*N *	#	*N *	#	Unadjusted TE	*p*-value	Adjusted TE^1^	*p*-value
Mean # of pregnancy complications listed	361	2.5	390	2.4	-0.19*	0.03	-0.24*	0.01
Mean # of delivery danger signs listed	215	1.7	253	1.6	-0.03	0.7	-0.02	0.87

**Table 7:** Part 2: Maternal knowledge of danger signs during pregnancy and post delivery amongst participants enrolled in PROMPTS and not-enrolled in PROMPTS at endline (among those who reported 'yes' for each respective category, linear regression results)

	Endline - Not Enrolled		Endline - Enrolled					
	*N *	#	*N *	#	Unadjusted TE	*p*-value	Adjusted TE^1^	*p*-value
Mean # of pregnancy complications listed	193	2.3	197	2.4	0.14	0.22	0.13	0.28
Mean # of delivery danger signs listed	127	1.7	126	1.5	-0.21	0.09	-0.29*	0.02
1. Controlled for age, wealth, marital status, sub-county, participant type, education, and employment status								
* *p*<0.05, ** *p*<0.01, *** *p*<0.001								

### Postnatal-newborn care seeking and knowledge

Outcomes related to PNC, newborn care seeking (Tables 8 and 9), and newborn care knowledge (Tables 10 and 11) were assessed among all participants with an infant 0—11 months old. There were significant improvements to care-seeking post-delivery with more mothers at endline reporting being checked by a provider compared to baseline (49% versus 65%; aOR = 1.88, *p*-value =  < 0.001). Women were also more likely to report seeking care within the nationally recommended time period of six days postpartum (51% versus 71%, aOR = 2.37, *p*-value =  < 0.001). Similarly, mothers at endline were more likely to report that their infants had received care relative to baseline (93% versus 97%; aOR = 1.85, *p*-value = 0.05). Infants were also more likely to receive care earlier, as the proportion of infants who were checked by a provider within 48 h (from 63 to 79%, *p*-value =  < 0.001) and within six days of birth increased at endline (from 69 to 84%; aOR = 2.56, *p*-value =  < 0.001).

**Table 8:** Part 1:  Postnatal and newborn care seeking amongst baseline and endline participants (Logisitc Regression Results)

	Baseline		Endline					
	*N *	*n *(%)	*N *	*n *(%)	Unadjusted OR	*p*-value	Adjusted OR^1^	*p*-value
Proportion of babies who were checked by a provider	491	457 ( 93% )	515	497 ( 97% )	2.05*	0.02	1.85*	0.05
Timing of baby’s first visit (amongst babies checked by a provider)								
Proportion of babies who were checked before 48 hours	468	295 ( 63% )	500	394 ( 79% )	2.18***	0	2.36***	0
Proportion of babies who were checked before 6 days	468	322 ( 69% )	500	419 ( 84% )	2.35***	0	2.56***	0
Proportion of mums who were checked by a provider	491	240 ( 49% )	515	333 ( 65% )	1.91***	0	1.88***	0
Timing of mum’s first visit (amongst mothers checked by a provider)								
Proportion of mothers who were checked before 48 hours	251	118 ( 47% )	336	222 ( 66% )	2.19***	0	2.22***	0
Proportion of mothers who were checked before 6 days	251	127 ( 51% )	336	237 ( 71% )	2.34***	0	2.37***	0

**Table 9:** Part 2:  Postnatal and newborn care seeking amongst participants enrolled in PROMPTS and not-enrolled in PROMPTS at endline (Logisitc Regression Results)

	Endline - Not Enrolled		Endline - Enrolled					
	*N *	*n* (%)	*N*	*n* (%)	Unadjusted OR	*p*-value	Adjusted OR^1^	*p*-value
Proportion of babies who were checked by a provider	278	263 ( 95% )	237	234 ( 99% )	4.45*	0.02	4.20*	0.03
Timing of baby’s first visit (amongst babies checked by a provider)								
Proportion of babies who were checked before 48 hours	265	202 ( 76% )	235	192 ( 82% )	1.39	0.14	1.56	0.06
Proportion of babies who were checked before 6 days	265	215 ( 81% )	235	204 ( 87% )	1.53	0.09	1.72*	0.04
Proportion of mums who were checked by a provider	278	167 ( 60% )	237	166 ( 70% )	1.55*	0.02	1.52*	0.03
Timing of mum’s first visit (amongst mothers checked by a provider)								
Proportion of mothers who were checked before 48 hours	169	109 ( 64% )	167	113 ( 68% )	1.15	0.54	1.18	0.51
Proportion of mothers who were checked before 6 days	169	118 ( 70% )	167	119 ( 71% )	1.07	0.77	1.03	0.91

1. Controlled for age, wealth, marital status, sub-county, participant type, education, and employment status

* *p*<0.05, ** *p*<0.01, *** *p*<0.001

**Table 10:** Part 1: Maternal knowledge - postnatal care for babies amongst baseline and endline participants (Logistic Regression Results)

	Baseline		Endline					
	*N *	*n* (%)	*N *	*n* (%)	Unadjusted OR	*p*-value	Adjusted OR^1^	*p*-value
Proportion of mothers who reported knowing about exclusive breastfeeding for 6 months	491	382 ( 78% )	515	425 ( 83% )	1.35	0.06	1.38*	0.05
Proportion of mothers who knew that they should keep the baby clothed or covered as much as possible at all time	491	377 ( 77% )	515	384 ( 75% )	0.89	0.41	0.91	0.54
Proportion of mothers who knew that they should avoid putting the baby on any cold or wet surface	491	194 ( 40% )	515	178 ( 35% )	0.81	0.1	0.82	0.15
Proportion of mothers who knew that they should avoid bathing the baby during the first six hours of life	491	71 ( 14% )	515	50 ( 10% )	0.64*	0.02	0.63*	0
Proportion of mothers who knew that they should wash hands with clean water and soap before and after handling the cord stump	491	112 ( 23% )	515	138 ( 27% )	1.24	0.14	1.32	0.07
Proportion of mothers who keep the cord stump exposed to air or loosely covered with clean clothes	491	70 ( 14% )	515	83 ( 16% )	1.16	0.41	1.16	0.43
Proportion of mums who know to avoid applying unclean substances on the cord stump	491	78 ( 16% )	515	93 ( 18% )	1.17	0.36	1.1	0.57
Proportion of mums who know to avoid covering the cord stump with bandages	491	41 ( 8% )	515	50 ( 10% )	1.18	0.45	1.15	0.54
Proportion of mums who know to hold pre-term baby skin-to-skin with mother	491	37 ( 8% )	515	23 ( 4% )	0.57*	0.04	0.51*	0.02
Proportion of mothers who reported knowing any danger signs for baby after delivery (Limited to mothers with a child aged 0-6 months)	285	206 ( 72% )	280	200 ( 71% )	0.96	0.82	0.88	0.52
Proportion of mothers who reported knowing any danger signs for the mom after delivery (Limited to mothers with a child aged 0-6 months)	285	172 ( 60% )	280	178 ( 64% )	1.15	0.43	1.13	0.5

**Table 11:** Part 2: Maternal knowledge - postnatal care for babies amongst participants enrolled in PROMPTS and not-enrolled in PROMPTS at endline (Logistic Regression Results)

	Endline - Not Enrolled		Endline - Enrolled					
	*N *	*n* (%)	*N *	*n* (%)	Unadjusted OR	*p*-value	Adjusted OR^1^	*p*-value
Proportion of mothers who reported knowing about exclusive breastfeeding for 6 months	278	232 ( 83% )	237	193 ( 81% )	0.87	0.55	0.83	0.44
Proportion of mothers who knew that they should keep the baby clothed or covered as much as possible at all time	278	200 ( 72% )	237	184 ( 78% )	1.35	0.14	1.33	0.18
Proportion of mothers who knew that they should avoid putting the baby on any cold or wet surface	278	93 ( 33% )	237	85 ( 36% )	1.11	0.57	1.19	0.36
Proportion of mothers who knew that they should avoid bathing the baby during the first six hours of life	278	30 ( 11% )	237	20 ( 8% )	0.76	0.37	0.74	0.35
Proportion of mothers who knew that they should wash hands with clean water and soap before and after handling the cord stump	278	75 ( 27% )	237	63 ( 27% )	0.98	0.92	0.99	0.96
Proportion of mothers who keep the cord stump exposed to air or loosely covered with clean clothes	278	46 ( 17% )	237	37 ( 16% )	0.93	0.77	0.92	0.72
Proportion of mums who know to avoid applying unclean substances on the cord stump	278	45 ( 16% )	237	48 ( 20% )	1.31	0.23	1.22	0.41
Proportion of mums who know to avoid covering the cord stump with bandages	278	24 ( 9% )	237	26 ( 11% )	1.3	0.37	1.3	0.39
Proportion of mums who know to hold pre-term baby skin-to-skin with mother	278	17 ( 6% )	237	6 ( 3% )	0.4	0.06	0.31*	0.02
Proportion of mothers who reported knowing any danger signs for baby after delivery (Limited to mothers with a child aged 0-6 months)	143	90 ( 63% )	137	110 ( 80% )	2.40**	0	2.21**	0.01
Proportion of mothers who reported knowing any danger signs for the mom after delivery (Limited to mothers with a child aged 0-6 months)	143	83 ( 58% )	137	95 ( 69% )	1.64	0.05	1.62	0.07

1. Controlled for age, wealth, marital status, sub-county, participant type, education, and employment status

* *p*<0.05, ** *p*<0.01, *** *p*<0.001

There were few observed changes in knowledge across the majority of newborn care practices (Table 10 and 11). Among the changes that were found, mothers were more likely to report knowledge of exclusive breastfeeding in the first six months of life (78% versus 83%, aOR = 1.38, *p*-value = 0.05). Mothers were *less* likely to report knowing that they should avoid bathing their infant in the first six hours of life (14% versus 10%; aOR = 0.63; *p*-value =  < 0.001). Mothers were also *less* likely at endline to report knowing to practice skin-to-skin with pre-term babies (8% versus 4%; aOR = 0.51, *p*-value = 0.02). A small but statistically significant decrease in knowledge of post-delivery danger signs for babies was found between baseline and endline (Tables 12 and 13), as the mean number of danger signs listed without prompting decreased (2.4 versus 2.1, b = -0.27, *p*-value = 0.04).

**Table 12:** Part 1:  Maternal knowledge of danger signs post delivery amongst baseline and endline participants (amongst those who reported 'yes' for each respective category,  linear regression results)

	Baseline		Endline					
	*N *	#	*N *	#	Unadjusted TE	*p*-value	Adjusted TE^1^	*p*-value
Mean # of baby post-delivery danger signs listed (Limited to mothers with a child aged 0-6 months)	206	2.4	200	2.1	-0.22	0.08	-0.27*	0.04
Mean # of mom post-delivery danger signs listed (Limited to mothers with a child aged 0-6 months)	172	1.8	178	1.7	-0.12	0.29	-0.12	0.31

**Table 13:** Part 2: Maternal knowledge of danger signs post delivery amongst participants enrolled in PROMPTS and not-enrolled in PROMPTS at endline (among those who reported 'yes' for each respective category, linear regression results)

	Endline - Not Enrolled		Endline - Enrolled					
	*N *	#	*N *	#	Unadjusted TE	*p*-value	Adjusted TE^1^	*p*-value
Mean # of baby post-delivery danger signs listed (Limited to mothers with a child aged 0-6 months)	90	2.3	110	2.0	-0.29	0.07	-0.33*	0.04
Mean # of mom post-delivery danger signs listed (Limited to mothers with a child aged 0-6 months)	83	1.8	95	1.6	-0.17	0.3	0.2	0.21

1. Controlled for age, wealth, marital status, sub-county, participant type, education, and employment status

* p<0.05, ** *p*<0.01, *** *p*<0.001

### PROMPTS Comparison Results

#### Participant and PROMPTS enrollment

An analysis of mothers on PROMPTS compared to those not on PROMPTS included 738 endline participants from Mathare and Kawangware in Nairobi County. Of the 738 endline participants, 331 (45%) were enrolled in the PROMPTS program (Fig. 2). Table 14 uses logistic regression to analyze the association between enrollment and participant demographic factors. There were higher odds of PROMPTS enrollment for participants who were older (aOR = 1.04, *p*-value = 0.01), unemployed (aOR = 1.45, *p*-value = 0.03), received a college / tertiary level education (aOR = 1.70, *p*-value = 0.03) relative to primary education, and who were in the higher wealth category (aOR = 1.84, *p*-value =  < 0.001).

**Table 14:** Unadjusted and adjusted odds ratios for being enrolled in PROMPTS amongst endline users

	*n *	Unadjusted OR	*p*-value		Adjusted OR^1^	*p*-value
Age	738	1.03*	0.04	736	1.04*	0.01
Unemployed	738	1.3	0.1	736	1.45*	0.03
Higher Wealth	738	1.96***	0	736	1.84***	0
Single	738	0.68*	0.02	736	0.81	0.23
Duration in ward	736	0.98	0.1	736	0.98	0.09
Education (reference = Primary)	738					
- Secondary		1.17	0.35	736	1.29	0.15
- Tertiary / College		1.43	0.11	736	1.70*	0.03
Participant Type (reference = ANC)	738					
- Have a child 0-6 months		1.31	0.14	736	1.24	0.25
- Have a child 7-11 months		1.01	0.97	736	1.11	0.58

1. Controlled for age, wealth, marital status, sub-county, participant type, education, and employment status

* p<0.05, ** *p*<0.01, *** *p*<0.001

User activity (defined as having at least one message sent by the participant to the PROMPTS system) was high among those enrolled, as 287 enrolled participants (87%) sent in a message to the system (Fig. 2). User activity was higher amongst mothers who were in the PNC period relative to those in the ANC period, as 226 participants with an infant 0–11 months (95%) had sent in at least one message into PROMPTS. Table 15 uses logistic regression to analyze the association between user activity amongst those enrolled in the PROMPTS program. Participant type was the only significant factor that was found to increase the odds of user activity, with there being increased activity among participants with an infant between 0 to 6 months (aOR = 6.94, *p*-value =  < 0.001) and those with infants between 7 to 11 months (aOR = 27.89, *p*-value =  < 0.001).

**Table 15:** Unadjusted and adjusted odds ratios for active PROMPTS amongst endline users

	*n*	Unadjusted OR	*p*-value	*n*	Adjusted OR^1^	*p*-value
Age	331	0.98	0.56	330	1.01	0.71
Employed	331	1.19	0.62	330	1.41	0.42
Status	331	1.18	0.64	330	0.86	0.73
Single	331	1.29	0.5	330	1.14	0.77
Duration in ward	330	1.02	0.5	330	1.02	0.52
Education (reference = Primary)	331					
- Secondary		1.92	0.07	330	1.98	0.11
- Tertiary / College		1.13	0.79	330	1.68	0.33
Participant Type (reference = ANC)	331					
- Have a child 0-6 months		6.66***	0	330	6.94***	0
- Have a child 7-11 months		25.97***	0	330	27.89***	0

1. Controlled for age, wealth, marital status, sub-county, participant type, education, and employment status

* *p*<0.05, ** p<0.01, *** *p*<0.00

### Antenatal knowledge and care-seeking

When comparing outcomes amongst participants enrolled in PROMPTS versus participants not enrolled in PROMPTS, there were improvements seen in ANC care-seeking and knowledge levels (Tables 2 and 3). Participants enrolled in PROMPTS were more likely to attend ANC compared to participants not enrolled in PROMPTS (96% versus 89%; aOR = 2.28 p-value = 0.05), but there were no significant differences found in the proportion of participants with at least four ANC visits.

A statistically significant increase in knowledge levels was observed amongst those enrolled in PROMPTS when it came to knowledge of complications during pregnancy (Tables 4 and 5). Participants who were enrolled in PROMPTS were more likely (86% versus 71%, aOR = 2.20, *p*-value =  < 0.01) to report knowing at least one sign of a pregnancy complication. A small but statistically significant difference in knowledge of delivery danger signs was found between participants not enrolled and enrolled in PROMPTS (Tables 6 and 7), as the mean number of delivery danger signs listed decreased (1.7 versus 1.5, b = -0.29, *p*-value = 0.02).

### Postnatal-newborn care seeking and knowledge

There were some improvements seen in postnatal care-seeking (Tables 8 and 9) and knowledge levels (Tables 10, 11, 12 and 13) amongst participants enrolled in PROMPTS. PROMPTS participants were more likely to report seeking care post-delivery (70% versus 60%, aOR = 1.52, *p*-value = 0.03). Similarly, mothers enrolled in PROMPTS were more likely to report their infants received care (99% versus 95%, aOR = 4.20, *p*-value = 0.03). A statistically significant difference in timeliness was not observed amongst mothers enrolled in PROMPTS and their infants.

Similar to the pre-post comparison, no changes in knowledge were observed across the majority of newborn care practices when comparing PROMPTS enrolled versus not-enrolled women (Tables 10 and 11); however, mothers enrolled in PROMPTS were more likely to report knowing any danger signs for their baby after delivery (80% versus 63%, aOR = 2.21, *p*-value =  < 0.01). Mothers enrolled in PROMPTS were less likely to report knowing to practice skin-to-skin with pre-term babies (3% versus 6%, aOR = 0.31, *p*-value = 0.02).

## Discussion

This research documents changes in care-seeking patterns and knowledge levels among pregnant and postnatal women living in two informal settlements in Nairobi, Kenya following the introduction of a digital health tool. This research also extends the PROMPTS body of evidence to informal urban settlements, which shows that when applied to an informal settlement context, the digital health tool, PROMPTS, has high rates of enrollment and activity amongst specific populations. PROMPTS is also associated with further improvements in specific aspects of maternal knowledge of danger signs and antenatal and postnatal careseeking for mothers and infants.

With respect to exploring changes due to the broader KAM intervention, there were improvements from baseline to endline with respect to mothers and infants who were checked by a provider post-delivery and the timeliness of these checks. While some of these improvements are associated with the broader KAM intervention (which includes PROMPTS), there is a possibility that the COVID-19 pandemic could have reduced care-seeking at baseline [20]. The pre-post comparison of the broader KAM intervention also found a small decrease in the proportion of mothers who reported knowing that they should hold pre-term babies skin-to-skin with the mother. One possible explanation for this could be the way that the question was asked leading to recall bias, as moms were asked to list practices and danger signs from memory rather than identifying whether a specific newborn care practice or danger sign was valid. At the same time, the recall period may have been too long, for example, mothers with infants 6–11 months may be less likely to recall specific newborn care taking practices.

This research also provides insights on the profiles of participants in an informal settlement that are most likely to engage with a digital health tool such as the PROMPTS platform. Enrollment was higher amongst participants that were in the ‘higher wealth’ segment, who were slightly older, had completed college / tertiary education, and were unemployed. Some of these findings are consistent with other mHealth interventions that have found factors like education can influence uptake of mHealth interventions. It also provides valuable perspectives and insights on groups that will need additional targeting to ensure that future versions of PROMPTS can successfully enroll and support additional participants [21]. Higher enrollment amongst those who were ‘higher wealth’ could be due to the fact that populations in this wealth category are more likely to have access to a phone [22]. PROMPTS user engagement was extremely high amongst those enrolled, with 89% of all participants sending in at least one message to the system, and with user engagement being higher amongst PNC mothers. Higher engagement in the PNC period is expected since these mothers, if enrolled during an ANC visit, would have had a longer time on the platform and had to elect to continue to stay on the platform post-delivery.

When comparing utilization and care-seeking and knowledge outcomes amongst those enrolled and not-enrolled in PROMPTS at endline, there were improvements in some aspects of knowledge and care-seeking. An improvement in care-seeking in the PNC period was observed for both mothers and infants. The proportion of mothers being checked by providers increased 1.5 times amongst mothers enrolled in PROMPTS. There was an increase in ANC care seeking amongst mothers enrolled in PROMPTS. This increase is partly driven by the fact that most participants who are enrolled in PROMPTS would have had to present at a facility for ANC in order to be enrolled; however, the method of peer enrollment could also have contributed to this increase. No statistically significant changes were observed in the proportion of mothers completing four ANC visits. In general, these outcomes were already higher in the study area (70% of mothers completing at least four ANC visits at baseline), relative to studies that have found mHealth interventions effective at increasing ANC visits [23].

PROMPTS users also showed an increase in knowledge levels for pregnancy complications, which is a core feature of the educational component of the PROMPTS platform. This is consistent with prior research on the PROMPTS platform [16]. It is worth noting that there was a decline observed in knowledge levels for some newborn care practices and for the mean number of delivery danger signs listed, albeit small in magnitude. Similar to the baseline and endline comparisons, it is suspected that recall bias may have affected this outcome.

These learnings on PROMPTS user profiles, suggests that future expansion of PROMPTS in informal settlements should target the unreached population, namely, younger mothers who have primary education. Additional research to understand why enrollment was not as high amongst these groups considering demographics, phone ownership, and other factors would help to drive expanded coverage. Understanding patterns of phone ownership within informal settlements in Kenya may also help with understanding enrollment and activity drivers, since mHealth interventions can have better outcomes when beneficiaries own their own phone [23].

This study provides important implications for ongoing digital health interventions that are targeted towards antenatal and postnatal mothers in informal settlements. Overall, both the high rates of enrollment and user activity contribute to the evidence of the potential effectiveness of using digital health tools to improve antenatal and postnatal outcomes in informal settlements. At the same time, it also makes it clear that technology solutions need to be carefully targeted to the unique populations they serve in order to yield the best outcomes and maximize coverage and adoption. In the future, targeting and refining enrollment efforts for PROMPTS to reach populations that were less likely to enroll during this study will be needed. For example, additional research can be conducted with those who did not enroll in the PROMPTS program to understand if additional improvements can be made and what barriers exist to enrollment. Similarly, additional research can be conducted with study participants enrolled in PROMPTS, to see how what improvements may be needed to drive behavior change and knowledge transfer more consistently across maternal and newborn health outcomes for informal settlement populations. User activity is high amongst those who were enrolled in the platform, and there were changes observed in some areas of care seeking and knowledge, suggesting that there is an opportunity to use PROMPTS to address other MNCH outcomes. However, additional research is needed in order to understand how much of these outcomes can be improved by PROMPTS refinement and what is only addressable through removing systemic barriers for access to care. In considering these findings, it is essential to contextualize them within the broader landscape of mhealth in Kenya. This study contributes valuable insights aligned with the existing literature on digital health initiatives in the country and strengthens the case for the possible benefits of utilizing digital health tools to enhance prenatal and postnatal care in informal settlements. It also highlights the need to carefully tailor technological solutions to specific populations. This focused strategy is essential for achieving the best results and increasing acceptance and coverage. The challenges and successes observed in informal settlements offer a nuanced understanding of the applicability and potential impact of mhealth interventions, further enriching the ongoing discourse on advancing healthcare through technology in Kenya.

### Limitations

This study had a few limitations. The implementation of this study took place during the height of the COVID-19 pandemic in Kenya, resulting in 1) a shift in surveying approach and 2) higher than normal movement within the study area. The study was originally meant to use in-person household surveys and a survey tool was designed accordingly with that approach in mind. Phone surveys are a viable and effective alternative to household surveys; however, they should be no longer than 15–30 min to avoid respondent fatigue. The baseline and endline surveys were 60 min in length which could have led to some respondent fatigue [24].

In addition, this was a pre-post study which runs the risk of being affected by changes to the study population in between the pre and post period. Due to the COVID-19 pandemic and the transitory nature of populations in informal settlements, we suspect that there was higher than normal migration out of the study area to rural areas by less wealthy households due to the lack of employment opportunities in Nairobi at the time. This resulted in differences between the baseline and endline populations; however, these were controlled for in the analysis.

We also included an analysis of the characteristics of the population that had been enrolled in PROMPTS compared to those who were not enrolled. The study was not intentionally designed to measure these outcomes, and the sample size from this group (*n* = 753) may not have been sufficient to evaluate our outcomes.

In addition, there could have been recall bias amongst participants when they were asked to list danger signs, pregnancy complications, and newborn care practices. Our hypothesis is that mothers may be likely to identify an individual danger sign or newborn care practice when directly asked about the danger sign or newborn care practice, rather than being asked to list from memory all possible danger signs for pregnancy, delivery, and newborn care. While this approach may result in social desirability bias, this approach is likely closer to the goals of PROMPTS as the program is designed to enable mothers to recognize a danger sign when it happens, rather than be able to list them from memory.

## Conclusion

The implementation of the PROMPTS digital health platform in informal settlements led to some significant changes, albeit variable in effect size, for postnatal and newborn care seeking and maternal knowledge of pregnancy complications. At the same time, this research was able to identify women who are more likely to be enrolled and engage in a two-way SMS system in informal settlements. This research contributes to the growing body of evidence of mHealth and digital health interventions, especially in informal settlements. Further research is recommended to understand what changes can be made in PROMPTS implementation in informal settlements to address gaps in enrollment and drive improvements in maternal, newborn, and infant outcomes.

### Supplementary Information

**Supplementary Material 1.**

## Data Availability

The data that support the findings of this study are available upon request from the corresponding author SR. De-identified data will be made publicly available in the Development Data Library.

## Abbreviations

ANC: Antenatal Care
aOR: Adjusted Odds Ratio
KAM: Kuboresha Afya Mitaani
LMIC: Low Middle Income Countries
LQAS: Lot Quality Assurance Sampling
MNCH: Maternal Newborn and Child Health
PCA: Principal Component Analysis
PNC: Postnatal Care
PROMPTS: Promoting Mothers through Pregnancy and Postpartum Through SMS
SA: Supervision Area
SES: Socioeconomic Status
SMS: Short Message System
